# Identification, Phylogeny, and Expression Profiling of Pineapple Heat Shock Proteins (HSP70) Under Various Abiotic Stresses

**DOI:** 10.3390/ijms252413407

**Published:** 2024-12-14

**Authors:** Rui Xu, Fangjun Wei, Yanzhao Chen, Faiza Shafique Khan, Yongzan Wei, Hongna Zhang

**Affiliations:** 1 Key Laboratory of Quality Regulation of Tropical Horticultural Crop in Hainan Province, School of Tropical Agriculture and Forestry, Hainan University, Haikou 570228, China; 22210902000003@hainanu.edu.cn (R.X.); weifangjun1002@163.com (F.W.); 23210902000005@hainanu.edu.cn (Y.C.); faizakhan@webmail.hzau.edu.cn (F.S.K.); 2 School of Breeding and Multiplication, Sanya Institute of Breeding and Multiplication, Hainan University, Sanya 572025, China; 3 State Key Laboratory of Biological Breeding of Tropical Crops, Institute of Tropical Bioscience and Biotechnology, Chinese Academy of Tropical Agricultural Sciences, Haikou 571101, China

**Keywords:** *Ananas comosus*, abiotic stress, AcHSP70 family, phylogeny, gene expression

## Abstract

Pineapple (*Ananas comosus* (L.) Merr.) is an economically significant and delicious tropical fruit. Pineapple commercial production faces severe decline due to abiotic stresses, which affect the development and quality of pineapple fruit. Heat shock protein 70 (HSP70) plays an essential role in abiotic stress tolerance. However, the pineapple *HSP70* family identification and expression analysis in response to abiotic stresses has not been studied. To explore the functional role of *AcHSP70*, different abiotic stress treatments were applied to pineapple cultivar “Bali” seedlings. A total of 21 *AcHSP70* members were identified in the pineapple genome. The identified genes were classified into four subfamilies (I–IV) using phylogenetic analysis. The *AcHSP70* family is expressed under different stress conditions. Quantitative real time polymerase chain reaction (qRT-PCR) revealed the expression pattern of the *AcHSP70* family under cold, drought, salt, and heat stress. The expression level of genes such as *AcHSP70-2* increased under heat, cold, and drought stress, while the expression level of genes such as *AcHSP70-3* decreased under salt stress. Furthermore, the expression profile of *AcHSP70*s in different tissues and development stages was analyzed using transcriptome analysis. The *HSP70* genes exhibited unique expression patterns in pineapple tissue at different developmental stages. The study therefore provides a list of *HSP70* genes with substantial roles in abiotic stress response and valuable information for understanding *AcHSP70* functional characteristics during abiotic stress tolerance in pineapple.

## 1. Introduction

Pineapple (*Ananas comosus* (L.) Merr.) is a perennial monocot plant belonging to the Bromeliaceae family. Pineapple is commercially cultivated as the third most important tropical fruit in China [1]. Its rich nutritional profile, distinctive flavor, and appealing texture render it highly desirable among global consumers [2]. As an economically significant crop, pineapple substantially contributes to the economies of various regions and plays a critical role in international trade, ranking just behind bananas and mangoes in tropical fruit production [3,4]. However, pineapple cultivation faces substantial challenges due to changing climatic conditions in terms of growth and productivity [5].

External environmental factors or abiotic stresses are a serious threat to global food production and security [6,7]. Abiotic stresses severely damage the physiological aspects of pineapple, such as internal browning and photosynthesis rate. Prolonged drought and hot temperature conditions significantly reduce pineapple yields [8]. Drought stress adversely affects fruit yield by resulting in stunted pineapple growth [9]. Low temperature affects the development of flowers and fruit in pineapple [10]. Various studies have analyzed minimum temperature as a critical climate variable in pineapple fruit quality [11]. However, a rise in minimum temperature to above 26 °C caused a hinderance in flower induction [12]. Abiotic stresses affect the internal browning of pineapple by changing metabolic pathways and causing oxidative damage to the fruit. Low temperature causes brown patches, black hearts, and internal browning [13]. Salinity stress affects the chemical quality of pineapple fruits [14]. To cope with these damages, breeders are working on the development of stress-tolerant varieties of pineapple to meet commercial production targets. Heat shock proteins (HSPs) are molecular chaperones synthesized by plants in response to abiotic stresses. HSP proteins are divided into the following five families based on molecular weight: the HSP100/ClpB family, the HSP90 family, the HSP70/DnaK family, the chaperonin family (HSP60/GroEL), and the small heat shock protein (HSP) family [15,16]. HSP70 is the most ubiquitously expressed Adenosine Triphosphate-(ATP)-dependent chaperone, typically weighing between 68 and 78 kDa. It is recognized as the most conserved HSP, playing a crucial role in protein-folding processes in conjunction with other molecular chaperones across various cellular compartments [17,18]. HSP70 mitigates the drastic effect of abiotic stresses and increases plant adaptivity to changing environmental conditions [19,20,21]. The *AtHSP70-16* gene inhibits seed germination under cold conditions in *Arabidopsis*. *Arabidopsis hsp70-50* mutants showed resistance against drought stress and reduced tolerance against heat stress [22]. Potato (*Solanum tuberosum*) *StHSP70* genes are significantly expressed in response to cold, heat, and drought stress [23]. In wheat (*Triticum aestivum* L.), eight *TaHSP70* genes showed high expression patterns under drought and heat stress conditions [24]. Tobacco (*Nicotiana tabacum*) *NtHSP70* showed distinct expression functions in response to abiotic stresses [25]. Similarly, jujube (*Ziziphus jujuba*) *ZjHsp70* members showed a high expression pattern against drought [16] and salinity heat stress and showed a crucial role in abiotic stress tolerance. In Litchi (*Litchi chinensis*), all six members of the *LcHSP70* gene family exhibit differential responses to cold, heat, drought, and salt stress [15]. Overall, *HSP70* genes showed significant variations in expression patterns against abiotic stresses in plants.

The role of the *HSP70* gene family has been identified in many plants. However, the *HSP70* gene family has rarely been studied in pineapple. Therefore, the identification of *HSP70* genes in the pineapple genome, the gene structure, cis-regulatory elements, and gene expression patterns would be of great significance. We performed a comprehensive study of the pineapple *HSP70* gene family and their expression patterns under abiotic stress conditions. We used bioinformatics tools to analyze the conserved domain, gene structure, physiochemical properties, and evolutionary relationship. Further, *AcHSP70* gene expression was investigated under different stress conditions and different developmental stages in pineapple. Our results are relevant for pineapple development under abiotic stresses and elucidate the mechanisms by which pineapple adapts to abiotic stresses. The future functional characterization of the *AcHSP70* gene family against abiotic stresses is critical and mandatory for commercial crop breeding.

## 2. Results

### 2.1. Identification and Chromosomal Localization of AcHSP70 Family

Utilizing the proteins encoded by the *Arabidopsis AtHSP70* genes as bait, we employed BLASTp and HMM methods to search the pineapple genomic data. After eliminating redundant sequences, 21 *AcHSP70* genes were identified; they were designated as *AcHSP70-1* to *AcHSP70-21* according to their chromosomal positions, and their physicochemical properties were predicted (Table S1). *AcHSP70-5* exhibits the most extended protein sequence, comprising 896 amino acids and a molecular weight of 99.26 kDa, while *AcHSP70-18* has the shortest sequence, consisting of 120 amino acids and a molecular weight of 13.06 kDa. The predicted isoelectric points (pI) for *AcHSP70s* range from 5.06 (*AcHSP70-2*) to 9.26 (*AcHSP70-18*). Among the 21 AcHSP70 proteins, 19.05% (four proteins) possess instability coefficients exceeding 40, indicating that the majority are stable. The aliphatic index varies from 76.06 (*AcHSP70-25*) to 103.13 (*AcHSP70-7*), with an average of 86.02. Except for *AcHSP70-7* and *AcHSP70-18*, which are hydrophobic, the remaining 19 proteins display hydrophilicity indices below 0, categorizing them as hydrophilic. Predictions for subcellular localization reveal that AcHSP70 proteins are primarily located in the endoplasmic reticulum (six proteins), mitochondria (nine proteins), nucleus (two proteins), and chloroplasts (four proteins).

**Figure 1:**
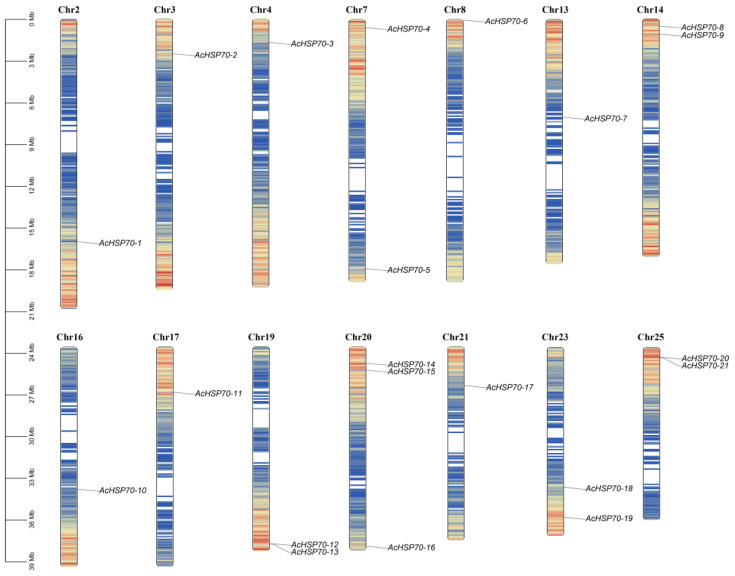
Chromosome distribution of AcHSP70 genes in pineapple. The *AcHSP70s* were located on Chr 2, 3, 4, 7, 8, 13, 14, 16, 17, 19, 20, 21, 22, and 25. Chr: chromosome. The ruler located on the left side represents the chromosome length and is shown in megabase (Mb).

The chromosomes, organized by descending length and labeled from Chromosome 1 to Chromosome 25, facilitated the mapping of *AcHSP70* genes based on precise gene location data. The mapping results indicate that the 21 *AcHSP70* genes are distributed across 14 distinct chromosomes (Figure 1). The distribution of *AcHSP70* genes across chromosomes exhibits significant variability with no discernible positional pattern. Notably, Chromosome 20 harbors three *AcHSP70* genes, whereas Chromosomes 2, 3, 4, 8, 13, 16, 17, and 21 each contain only one *AcHSP70* gene.

### 2.2. Phylogenetic Analysis of the AcHSP70 Family

**Figure 2:**
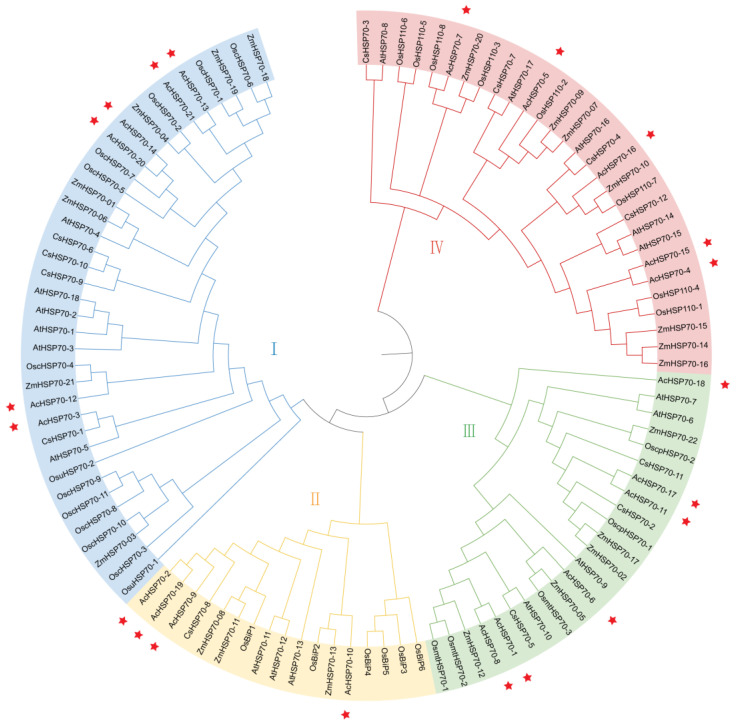
The phylogenetic analysis of AcHSP70 proteins with *Arabidopsis*, cucumber (*Cucumis sativus* L.), rice (*Oryza sativa* L.), and maize (*Zea mays* L.). The phylogenetic tree was made by using MEGA 11.0 software with the neighbor-joining (NJ) method, and the bootstrap replications were set to 1000 times. Different colors represent four groups (I–IV), and stars represent *AcHSP70s*.

To explore the evolutionary relationships in the pineapple HSP70 family, we incorporated 18 *Arabidopsis* AtHSP70 proteins, 12 cucumber CsHSP70 proteins, 32 rice OsHSP70 proteins, and 21 maize ZmHSP70 proteins to construct a phylogenetic tree, utilizing the neighbor-joining method. The analysis revealed that the AcHSP70 family is divided into four subfamilies (I–IV), comprising six, four, six, and five members, respectively, with an uneven distribution of AcHSP70 members within each subfamily (Figure 2). Furthermore, the *AcHSP70* genes in pineapple demonstrate significant similarity and evolutionary relationships with *HSP70* genes from monocotyledonous plants, such as rice and maize, while exhibiting reduced similarity with *HSP70* genes from dicotyledonous plants, including Arabidopsis and cucumber. This indicates that the biological functions of *HSP70* genes may be more comparable among monocotyledonous species.

### 2.3. Gene Structures and Conserved Motifs Analysis

**Figure 3:**
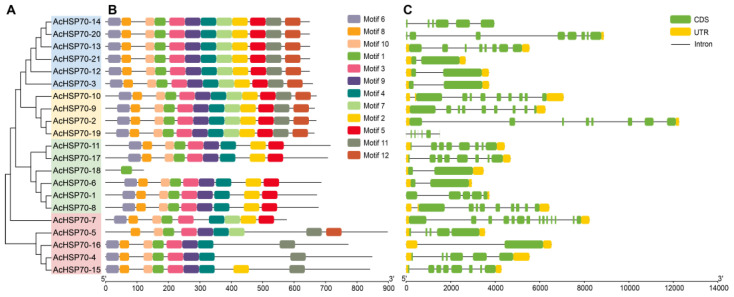
Gene structures and conserved motifs of *AcHSP70s*. (**A**) Different colors represent the four groups of *AcHSP70* genes (I–IV). (**B**) The motifs of AcHSP70 proteins are shown as colored boxes. (**C**) Gene structures of *AcHSP70* genes. The yellow blocks represent the coding sequence (CDS), the green blocks represent the untranslated region (UTR), and the black lines represent introns.

The HSP70 protein consists of three domains: an N-terminal nucleotide-binding domain (NBD) of approximately 44 kDa, a substrate-binding domain (SBD) of about 18 kDa, and a C-terminal substrate-binding domain measuring roughly 10 kDa [23,26,27]. To examine the sequence characteristics of AcHSP70 proteins, an analysis of their conserved motif composition was performed, leading to the identification of 12 distinct conserved motifs (Figure 3B and Figure S1). Among the 20 *AcHSP70* members, the number of motifs ranges from eight to twelve, with each subfamily displaying comparable gene structures. Notably, *AcHSP70-18* contains only a single motif. In order to study the structural characteristics of the *AcHSP70* gene family, intron and exon composition analysis was performed. The findings reveal considerable variability in the gene structures of *AcHSP70s*, with intron numbers ranging from 1 to 13 (Figure 3C). The member with the highest intron count is *AcHSP70-7*, which possesses 13 introns. Members within the same *AcHSP70s* subfamily demonstrate comparable intron–exon structural characteristics.

### 2.4. Cis-Acting Elements Analysis of AcHSP70s Family

**Figure 4:**
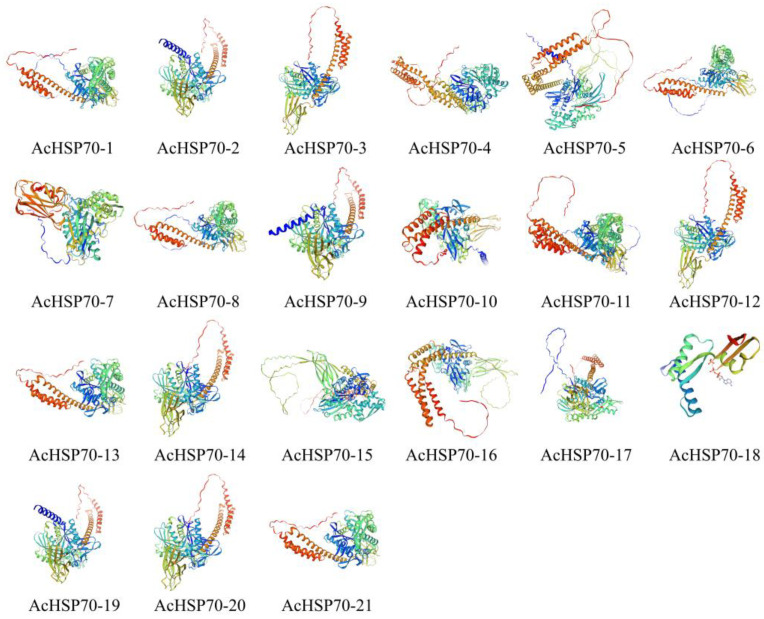
Three-dimensional structural analysis of *AcHSP70s*.

The predicted secondary structures of AcHSP70 proteins reveal that all 21 AcHSP70 variants comprise alpha helices, extended strands, and random coils (Table S2). The percentage of alpha helices varies between 34.96% and 45.67%, while random coils comprise 36.45% to 44.64% of the structure. Both elements are predominant in the AcHSP70 proteins, in contrast to extended strands, which are less abundant, accounting for 12.38% to 25.00%. Three-dimensional structural analyses suggest that members of the same subfamily exhibit more structural similarity (Figure 4).

**Figure 5:**
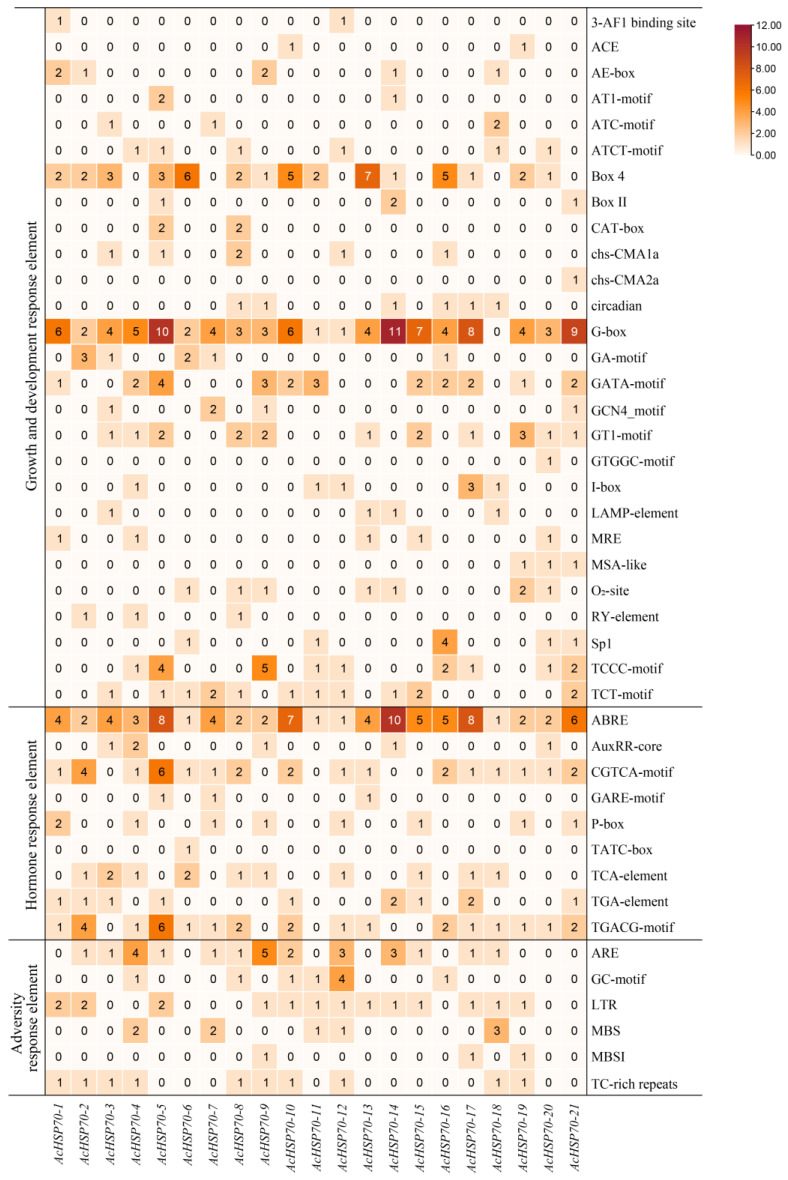
The *cis*-acting elements in promoters of *AcHSP70* genes. The amounts of *cis*-elements in *AcHSP70s* promoter regions were displayed in different colors and numbers in the grid.

Predicted analyses of the cis-acting elements in the promoter regions of *AcHSP70* genes reveal that all *AcHSP70* genes possess diverse quantities of these elements within the upstream 2000 bp of their promoters. These elements are classified into three primary categories: growth and development response elements, hormone response elements, and adversity response elements (Figure 5).

Among the plant growth and development response elements, a total of 27 types have been identified, including light response elements (e.g., Box 4, G-box, GATA-motif, TCCC-motif), elements linked to circadian rhythm regulation (circadian), and elements associated with cell structure (CAT-box, GCN4_motif, RY-element). The hormone response elements comprise nine types, encompassing abscisic acid response elements (ABRE), jasmonic acid methyl ester response elements (CGTCA-motif, TGACG-motif), auxin response elements (AuxRR-core, TGA-element), gibberellin response elements (GARE-motif, P-box, TATC-box), and salicylic acid response elements (TCA-element). Additionally, six types of adversity response elements are present, including drought-induced response elements (MBS, MBSI), cold response elements (LTR), hypoxia-specific induced response elements (GC-motif), anaerobically induced response elements (ARE), and defense and adversity response elements (TC-rich repeats). These findings indicate that *AcHSP70s* may play a pivotal role in the processes of growth and development, hormone response, and stress response in pineapple.

### 2.5. Intra-Species Synteny Analysis of the AcHSP70 Family

**Figure 6:**
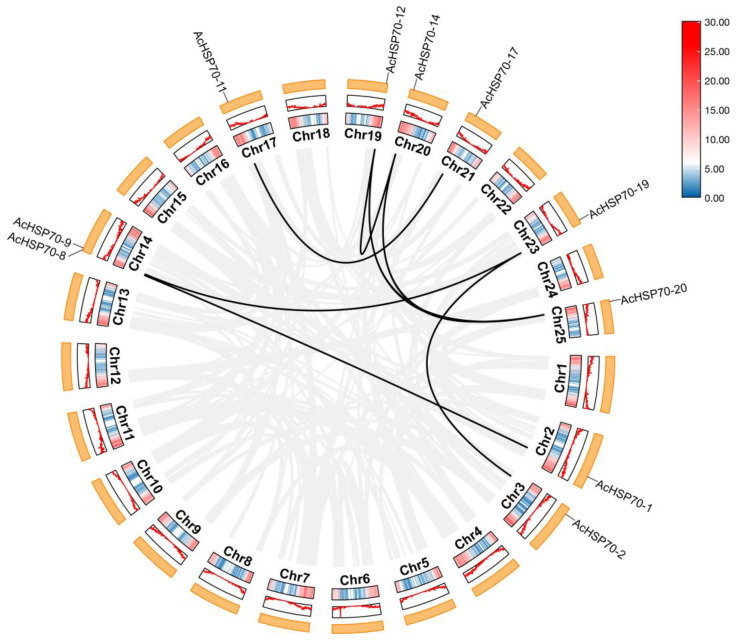
Intraspecies synteny analysis of *AcHSP70* genes. The black curve represents duplication events between *AcHSP70* genes. Chr 1–25: Chromosome 1–25.

**Figure 7:**
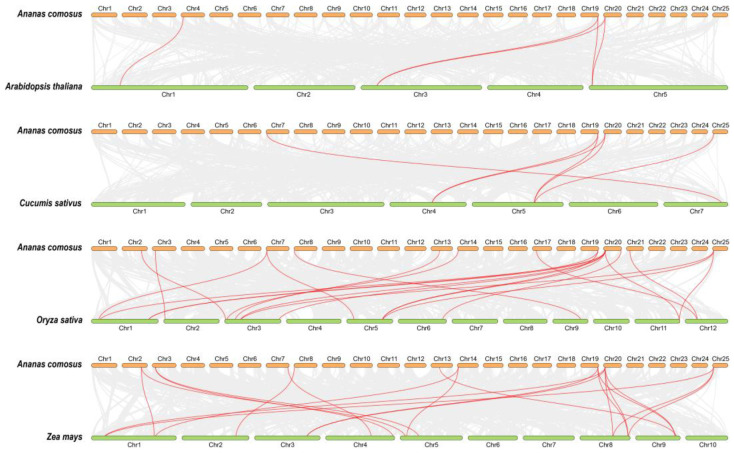
Collinearity of *HSP70* genes in pineapple. The gray line represents the collinearity of all the genes in the pineapple, and the red line represents the collinearity of the *AcHSP70* genes.

The analysis of intragenomic collinearity in *AcHSP70s* indicated the absence of tandem duplications or gene translocation events. Among the 21 *AcHSP70s*, the following seven pairs of segmental duplication genes were identified: *AcHSP70-1*/*AcHSP70-8*, *AcHSP70-2*/*AcHSP70-19*, *AcHSP70-9*/*AcHSP70-19*, *AcHSP70-11*/*AcHSP70-17*, *AcHSP70-12*/*AcHSP70-14*, *AcHSP70-12*/*AcHSP70-20*, and *AcHSP70-14*/*AcHSP70-20* (Figure 6). The quantities of syntenic *HSP70* genes between pineapple and the respective species, such as *Arabidopsis*, cucumber, rice, and maize, are five, six, twenty-one, and twenty, respectively (Figure 7). Notably, although not all *HSP70* genes in *Arabidopsis* and cucumber have homologous counterparts in pineapple, the majority of *HSP70* genes in rice and maize possess one or more homologs in pineapple. Intra-species synteny analysis suggests that the *AcHSP70* gene family in pineapple predominantly arises through segmental duplications. Meanwhile, inter-species synteny analysis indicates a closer evolutionary relationship between pineapple and the monocotyledon rice and maize.

### 2.6. Tissue-Specific Expression Pattern of AcHSP70s

**Figure 8:**
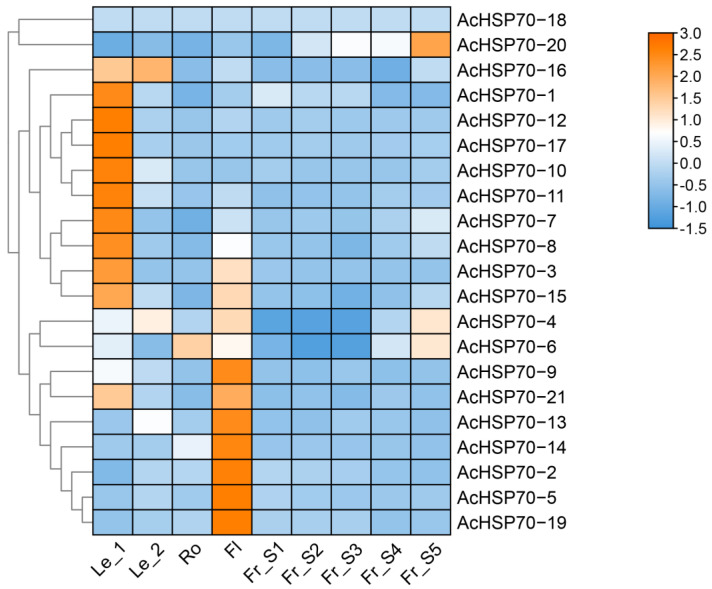
Expression profiles of *AcHSP70* family members in pineapple leaves with and without spines. Transcriptomic data (Le_1: Leaf apices; Le_2: Leaf base; Ro: Root; Fl: Flower; Fr: fruit) were analyzed using Log2(FPKM) values. The color scale on the right represents the relative expression level, from high (orange) to low (blue).

To explore the potential role of the *AcHSP70* gene family, transcriptome data from various pineapple tissues (leaves, roots, flowers, and fruits) were analyzed using FPKM (fragments per kilobase million) values. The results demonstrated significant variations in the expression of *AcHSP70* gene members across different pineapple tissues. In the leaf tip, 11 members, including *AcHSP70-1*, exhibit elevated expression levels, whereas only *AcHSP70-4* and *AcHSP70-16* were highly expressed at the leaf base. *AcHSP70-6* showed high transcription levels in the roots, and 10 *AcHSP70* members displayed high expression in flowers. Although most *AcHSP70s* exhibited low expression during fruit development, the expression levels of *AcHSP70-4*, *AcHSP70-6*, and *AcHSP70-20* increased progressively as the fruit matured (Figure 8).

### 2.7. AcHSP70 Family Expression Analysis Under Heat Stress

**Figure 9:**
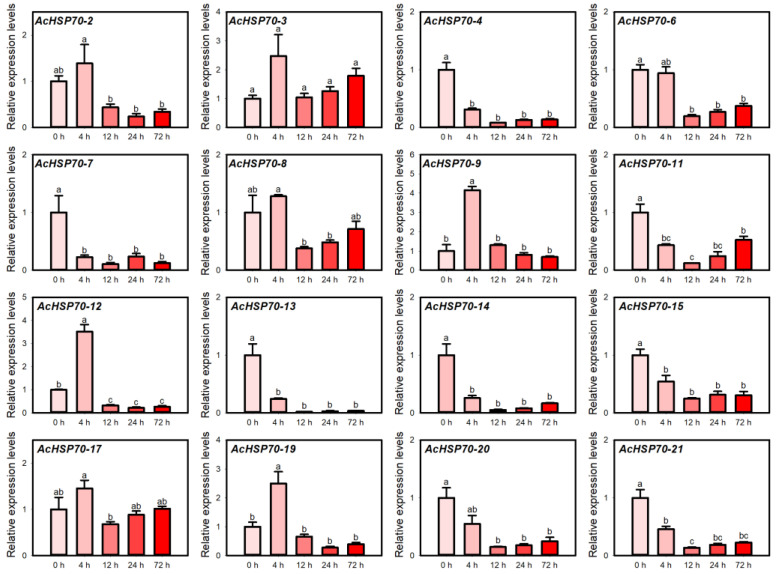
Expression levels of *AcHSP70* genes under 0 h control (CK), 4 h, 12 h, 24 h, and 72 h of heat stress treatment. Data are expressed as means ± SD (*n* = *3*). Different letters indicate significant differences between groups (*p* < 0.05).

Heat or high temperature can induce the accumulation of reactive oxygen species (ROS) within organelles, disrupting normal physiological and biochemical processes in plants and ultimately hindering their growth and development [28,29]. To investigate the role of the *AcHSP70* genes under abiotic stress, QRT-PCR was used to analyze expression patterns of AcHSP70 genes. As illustrated in Figure 9, *AcHSP70-1*, *AcHSP70-5*, *AcHSP70-10*, *AcHSP70-16*, and *AcHSP70-18* genes were undetectable, whereas the other 16 *AcHSP70* members were differentially expressed in response to heat stress. Expression levels of *AcHSP70-2*, *AcHSP70-9*, *AcHSP70-12*, *AcHSP70-17*, and *AcHSP70-19* displayed increasing and decreasing trends after 4 h of treatment. In contrast, the expression levels of the remaining 10 members, excluding *AcHSP70-3*, exhibited a decreasing and increasing trend.

### 2.8. AcHSP70 Family Expression Analysis Under Cold Stress

**Figure 10:**
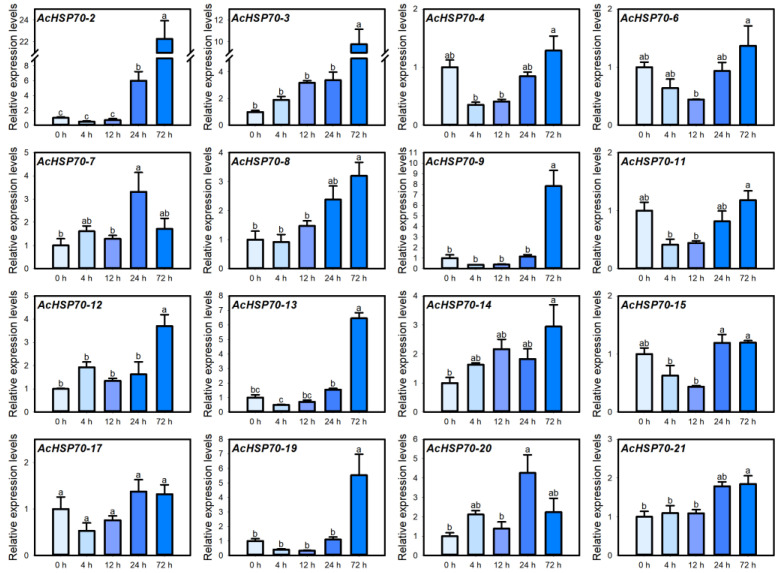
Expression levels of *AcHSP70* genes under 0 h control (CK), 4 h, 12 h, 24 h, and 72 h of cold stress treatment. Data are expressed as means ± SD (*n* = *3*). Different letters indicate significant differences between groups (*p* < 0.05).

Cold stress is an abiotic factor that significantly hampers plant growth and development. Extended exposure to cold can disrupt the physiological condition of plants, resulting in damage and potentially leading to mortality [30]. Under cold stress, the expression levels of genes such as *AcHSP70-2*, *AcHSP70-3*, *AcHSP70-8*, *AcHSP70-9*, *AcHSP70-12*, *AcHSP70-13*, *AcHSP70-14*, *AcHSP70-19*, and *AcHSP70-21* progressively increase with prolonged exposure, peaking at 72 h. In contrast, *AcHSP70-7* and *AcHSP70-20* display a “first increase, then decrease” pattern, with their maximum expression occurring 24 h before a subsequent decline (Figure 10).

### 2.9. AcHSP70 Family Expression Analysis Under Drought Stress

**Figure 11:**
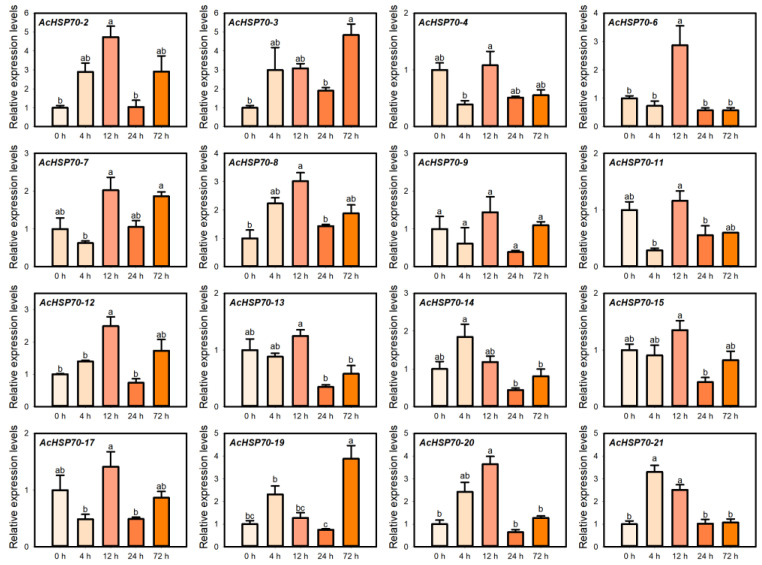
Expression levels of *AcHSP70* genes under 0 h control (CK), 4 h, 12 h, 24 h, and 72 h of drought treatment. Data are expressed as means ± SD (*n* = *3*). Different letters indicate significant differences between groups (*p* < 0.05).

Drought impairs plant root water absorption, subsequently affecting transpiration, photosynthesis, and respiration. Furthermore, it may cause the accumulation of toxic ions in plant tissues, hindering normal growth and development [31]. Under drought stress, the expression levels of *AcHSP70-2*, *AcHSP70-8*, *AcHSP70-12*, and *AcHSP70-20* increased progressively with treatment duration, peaking at 12 h, followed by a decline and a subsequent peak at 72 h. In contrast, *AcHSP70-14* and *AcHSP70-21* peaked at 4 h before gradually decreasing. *AcHSP70-3* and *AcHSP70-19* reached their maximum expression at 72 h, while *AcHSP70-6* exhibited its highest expression at 12 h (Figure 11).

### 2.10. AcHSP70 Family Expression Analysis Under Salt Stress

**Figure 12:**
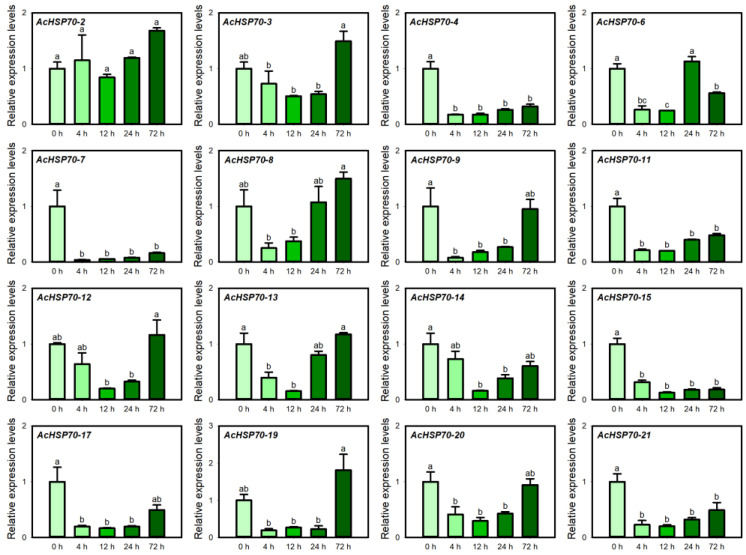
Expression levels of *AcHSP70* genes under 0 h control (CK), 4 h, 12 h, 24 h, and 72 h of salt stress treatment. Data are expressed as means ± SD (*n* = *3*). Different letters indicate significant differences between groups (*p* < 0.05).

Salt stress adversely impacts root development, affecting plant morphology and growth processes [32]. Under salt stress, the expression levels of all members demonstrated a pattern of “initial decrease followed by an increase”. Specifically, *AcHSP70-3*, *AcHSP70-8*, *AcHSP70-9*, *AcHSP70-12*, *AcHSP70-13*, *AcHSP70-19*, and *AcHSP70-20* exhibited their lowest expression at 4 or 12 h, followed by a gradual increase, peaking at 72 h. Similarly, *AcHSP70-4*, *AcHSP70-7*, *AcHSP70-11*, *AcHSP70-14*, *AcHSP70-15*, *AcHSP70-17*, and *AcHSP70-21* reached their minimum levels at 4 or 12 h before rising. In contrast, *AcHSP70-6* exhibited peak expression 24 h post-treatment (Figure 12).

## 3. Discussion

Abiotic stresses severely damage plant development and lead to a significant decline in commercial crop production. Abiotic stresses significantly reduced the growth and quality of pineapple fruit. Pineapple is an important commercial crop in tropical and subtropical areas of China. The pineapple industry in China totally depends on the introduction of foreign varieties such as Bali, Thornless Cocaine, and Shenwan. The Bali variety is estimated to have a cultivation area of around 75% in China [33]. This presents a significant challenge regarding the adaptation of foreign varieties to changing climatic conditions.

The current research work focused on the identification and expression profiling of the *HSP70* gene family under abiotic stresses. In this study, we identified 21 *AcHSP70* in the pineapple genome. The *AcHSP70* proteins exhibited a considerable variation in the amino acid count, relative molecular weight, and isoelectric point, which may indicate their functional diversity. Subcellular localization for *AcHSP70* proteins revealed their presence in the nucleus, endoplasmic reticulum, mitochondria, and chloroplasts. This distribution aligns with the predicted localization of *HSP70* proteins in other plants, such as rice and maize, implying that the pineapple *HSP70* gene family may share similar roles in growth development and abiotic stress responses observed in these crops.

Phylogenetic analysis is used to elucidate the evolutionary relationships among genes. Closely grouped genes tend to display analogous structures and functions [34]. This study developed a phylogenetic tree for the *HSP70* gene family in pineapple, comparing it with that of *Arabidopsis*, cucumber, rice, and maize based on their evolutionary relationships. The findings indicated that the 21 *AcHSP70s* members can be categorized into four subfamilies, aligning with the classification observed in rice and maize. Phylogenetic and gene structure analyses of *AcHSP70* proteins showed a high-similarity closed relationship with the subfamilies. The analysis of predicted subcellular localization showed that four members of subfamily II are localized in the endoplasmic reticulum and four members of subfamily III are localized in the mitochondria. Furthermore, *AcHSP70-11* and *AcHSP70-17* are localized in the chloroplast. The intron count in *AcHSP70* genes varies from 0 to 13, a range that parallels the *HSP70* structures observed in grapevine (*Vitis vinifera*) [35] and maize [36]. These results show high conservation between *HSP70* family members across various species.

Gene duplication is a critical mechanism in species evolution and the emergence of new genes, primarily occurring through three processes: segmental duplication, tandem duplication, and gene transposition. Notably, segmental and tandem duplications are the principal mechanisms driving the expansion of plant gene families [37]. In *AcHSP70s*, seven pairs of segmental duplication events have been identified, with no instances of tandem duplication or gene transposition observed. The *AcHSP70* family exhibits homologous relationships with one or more genes in monocotyledonous plants such as rice and maize. However, not all *HSP70* genes in dicotyledonous plants like *Arabidopsis* and cucumber demonstrate homologous relationships. This finding is consistent with their evolutionary relationships and suggests that the pineapple *AcHSP70* family may exhibit functional similarities to *HSP70* genes in monocots.

The promoter sequence provides the fundamental basis for the analysis of gene expression and regulation [36]. *Cis*-acting elements are integral to signal transduction, and the promoter region of the plant *HSP70* gene typically includes hormone and stress response elements [38]. This study reveals the presence of various cis-acting elements associated with plant growth, hormone response, and stress response in the upstream region of the *AcHSP70s* promoter sequence. Predominantly, the light response element G-box is most abundant, accompanied by significant occurrences of abscisic acid response elements (ABRE), cold response elements (LTR), and drought response elements (MBS), among others. It is well established that numerous plant hormones are critical for mediating responses to stressful environments. Abscisic acid (ABA) is essential for plant adaptation to abiotic stress. Consequently, it can be inferred that AcHSP70 proteins may be integral to various processes, including plant growth, hormone regulation, and stress response [39,40].

Plant *HSP70* genes have diverse responses to abiotic stress and induce tolerance in plants [41,42]. Under cold stress, the expression levels of several members, including *AcHSP70-2*, *AcHSP70-3*, and *AcHSP70-9*, exhibited an upward trend, reaching their peak at 72 h of treatment. This observation aligns with the expression patterns noted in cabbage leaves under similar cold stress conditions [43]. Under heat stress, the *AcHSP70* genes demonstrated two distinct expression patterns. The expression pattern of *AcHSP70-4*, *AcHSP70-6*, and *AcHSP70-7* decreased to their lowest point at 12 h post-treatment before gradually increasing. In contrast, *AcHSP70-2*, *AcHSP70-9*, *AcHSP70-12*, *AcHSP70-17*, and *AcHSP70-19* genes exhibited an initial rise after exposure to heat, peaking at 4 h, followed by a gradual decline. This observation parallels the expression patterns of *HSP70* genes in Chinese pepper subjected to heat stress [44]. *AcHSP70* gene responses to temperature exhibit substantial variability. Most members, including *AcHSP70-2*, *AcHSP70-3*, and *AcHSP70-9*, are primarily responsive to low temperatures. In contrast, *AcHSP70-2*, *AcHSP70-9*, *AcHSP70-12*, *AcHSP70-17*, and *AcHSP70-19* likely play critical roles in the pineapple’s adaptation to heat stress.

Under drought stress, *AcHSP70-2*, *AcHSP70-6*, *AcHSP70-8*, *AcHSP70-12*, *AcHSP70-13*, *AcHSP70-14*, *AcHSP70-20*, and *AcHSP70-21* showed the highest expression pattern under 4 to 12 h post-treatment before gradually decreasing. The ABA response element (ABRE) detects ABA signals, promoting the expression of related genes that enhance plant drought tolerance. Additionally, the MYB binding site (MBS) improves plant resilience under drought stress [45,46]. In this study, multiple cis-acting elements associated with abiotic stress were identified in the promoter region of the *AcHSP70s* gene. Notably, the ABA response element and MYB binding site may be pivotal in mediating the *AcHSP70s* gene’s response to drought stress.

In our study, the expression levels of *AcHSP70-3*, *AcHSP70-8*, *AcHSP70-9*, *AcHSP70-12*, *AcHSP70-13*, *AcHSP70-19*, and *AcHSP70-20* displayed a “decrease-then-increase” pattern, with their lowest expression observed at 4 or 12 h of treatment, followed by a gradual increase under salt stress treatment. These results align with the expression patterns observed in specific *TaHSP70s* genes in wheat subjected to salt stress [24]. These findings suggested a positive role of *AcHSP70* genes against abiotic stress response.

## 4. Materials and Methods

### 4.1. Plant Materials and Experimental Treatments

Three-month-old “Bali” pineapple tissue culture seedlings (dimensions: 3 × 6 cm, cultivated in MS medium) underwent several treatments for the experiment. Heat stress was imposed by transferring the plants to an incubator (model: PRX-450D) at 40.0 ± 1.0 °C, with a light intensity of 2600 lx, relative humidity of 60%, and a 12 h photoperiod. Cold stress was applied at 4.0 ± 1.0 °C under the same conditions. Drought stress was simulated using a 15% polyethylene glycol (PEG) 6000 solution, while salt stress was induced with a 200 mM NaCl solution. Leaf samples were collected at 0, 4, 12, 24, and 96 h post-treatment, with each treatment including three biological replicates. Samples were immediately frozen in liquid nitrogen and stored at −80 °C for analysis.

### 4.2. Identification of HSP70 Genes in Pineapple

The complete genome file of pineapple was downloaded from the pineapple genome database (https://ananas.watchbio.cn/file_download/download.php, accessed on 10 August 2024), while the *AtHSP70s* protein sequences were obtained from the TAIR database [47]. Using the bait sequence of *AtHSP70s*, TBtools (v2.110) software was utilized to perform blastp analysis on the HSP70 gene family in the pineapple genome [48]. The hidden Markov model (HMM) for the HSP70 protein domain (PF00012) was acquired from the Pfam database (http://pfam-legacy.xfam.org/, accessed on 10 August 2024) and employed to search the pineapple protein database, leading to the initial identification of candidate proteins [49].

The candidate protein sequences were assessed for conserved domains utilizing the NCBI Conserved Domain Database (CDD, https://www.ncbi.nlm.nih.gov/cdd, accessed on 14 August 2024) [50]. Redundant sequences were eliminated, yielding a final selection of 21 members of the *AcHSP70* family, designated *AcHSP70-1* to *AcHSP70-21* according to their chromosomal positions. The validated AcHSP70 protein sequences from pineapple were analyzed using the ProParam tool in ExPASy (https://web.expasy.org/protparam/, accessed on 21 August 2024) to determine their physicochemical properties. This analysis included predictions of amino acid (AA) length, molecular weight (MW), isoelectric point (PI), instability index, aliphatic index, and grand average of hydropathicity (GRAVY). Subcellular localization predictions were conducted using the Cell-PLoc 2.5 (http://www.csbio.sjtu.edu.cn/bioinf/plant-multi/, accessed on 22 August 2024) online tool.

### 4.3. Chromosome Localization Phylogenetic Relationships

Data on chromosome lengths, chromosomal locations, and the starting positions of the *AcHSP70* genes were extracted from the pineapple genome database (https://ananas.watchbio.cn/file_download/download.php, accessed on 10 August 2024). The pineapple chromosomes were organized in descending order by length, designated as Chr1 to Chr25. Subsequently, the chromosomal localization map for *AcHSP70s* was created by utilizing the Gene Location Visualize function in TBtools (v2.110) [51].

To investigate the phylogenetic relationships between AcHSP70 proteins and their functional role during stresses in pineapple, the phylogenetic tree was constructed using the protein sequences of 21 *AcHSP70s* from pineapple, 18 *AtHSP70s* from *Arabidopsis* [52], 12 CsHSP70s from cucumber [53], 32 OsHSP70s from rice (*Oryza sativa* L.) [54], and 21 ZmHSP70s from maize (*Zea mays* L.) [32]. HSP70 family protein sequences were obtained from TAIR (https://www.arabidopsis.org/, accessed on 10 August 2024), Rice genome annotation project (RGAP) (http://rice.uga.edu/, accessed on 22 August 2024), TFGD (http://ted.bti.cornell.edu/, accessed on 22 August 2024), and ZEAMAP (https://db.cngb.org/zeamap/, accessed on 22 August 2024). The sequences were aligned with ClustalX (v. 1.83), and a phylogenetic tree was constructed using MEGA (v. 11.0), incorporating 1000 bootstrap replicates for validation [55]. The phylogenetic tree was refined using the iTOL (https://itol.embl.de/, accessed on 23 August 2024) online tool [56].

### 4.4. Analysis of Gene Structures and Conserved Motifs

The gene structure of *AcHSP70s*, encompassing the locations of exons, introns, and UTRs (untranslated regions), was analyzed using the GFF (Generic Feature Format) file of the pineapple genome via the GSDS 2.0 (https://gsds.gao-lab.org/Gsds_help.php, accessed on 25 August 2024) online platform [57]. Conserved motifs of the *AcHSP70s* protein were analyzed using the MEME (https://meme-suite.org/meme/tools/meme, accessed on 26 August 2024) web platform, with the motif count specified as 12. The findings were subsequently visualized utilizing the Visualize MEME/MAST Motif Pattern function in TBtools (v2.110).

### 4.5. Prediction of AcHSP70 Structure and Promoter Cis-Acting Elements

A new method, self-organized prediction (SOPM), was used to predict the secondary structure of proteins. This method accurately predicts amino acids for three three-state descriptions of the secondary structure. The AcHSP70 proteins’ structure was used for prediction and analyzed via the SOPMA (https://npsa.lyon.inserm.fr/cgi-bin/npsa_automat.pl?page=/NPSA/npsa_sopma.html, accessed on 27 August 2024) web platform [58]. Geno3D: Automatic-based comparative modeling of protein three-dimensional (3-D) structure features was used, and AcHSP70 protein sequences were pasted one by one, according to expectation value (-e, real) 1.0. Further, AcHSP70 three-dimensional conformation was modeled using the SWISS (https://swissmodel.expasy.org/interactive, accessed on 27 August 2024) online tool [59]. Cis-regulatory elements were analyzed to understand the regulatory mechanisms of AcHSP70 genes. A 2kb upstream promoter sequence situated above the coding region was examined using the PlantCARE (https://bioinformatics.psb.ugent.be/webtools/plantcare/html/, accessed on 30 August 2024) web resource [60]. The results were visualized with the HeatMap plugin in TBtools (v2.110) [61].

### 4.6. Gene Replication and Collinearity Analysis of AcHSP70 Genes Family

Chromosome location data were obtained from the pineapple genome and annotation files, and the circular gene view function of TBtools (v2.110) was utilized to visualize the *AcHSP70s* gene location information. This approach enabled an intraspecific collinearity analysis of the pineapple *HSP70* genes. Genomic and annotation files for *Arabidopsis*, cucumber, rice, and maize were retrieved from the Ensembl database (http://plants.ensembl.org/index.html, accessed on 21 August 2024) [52]. TBtools (v2.110) was subsequently employed to analyze and illustrate segmental duplication events among these four species in relation to pineapple, thereby facilitating an interspecific collinearity analysis [48].

### 4.7. Expression Pattern Analysis of AcHSP70 Genes

Transcriptome data from various tissues and developmental stages of pineapple have been published, with the data utilized in this study sourced from public databases (http://pineapple.zhangjisenlab.cn/pineapple/html/mRNA.html, accessed on 2 September 2024) [62]. We selected transcriptome data corresponding to the pineapple leaf tip, leaf base, root, flower, and fruit developmental stages. Utilizing TBtools (v2.110), we generated a heatmap to visualize the expression patterns of *AcHSP70* genes.

### 4.8. Expression Analysis of the AcHSP70 Family Under Various Abiotic Stress Conditions

Total RNA was extracted from preserved ‘Bali’ pineapple leaves using the Fast Universal Plant RNA Extraction Kit 3.0 (Beijing Huayueyang Biotechnology Co., Ltd., Beijing, China). The concentration and purity of the extracted RNA were evaluated with a NanoDrop Lite spectrophotometer (Thermo Fisher Scientific, Waltham, MA, USA), yielding an OD_260/280_ ratio between 1.8 and 2.0. Total RNA was reverse transcribed into cDNA using the RevertAid First Strand cDNA Synthesis Kit (Thermo Fisher Scientific, Waltham, MA, USA) for fluorescence quantitative analysis of *AcHSP70s*. Quantitative real-time PCR (qRT-PCR) primers were designed using the Batch q-PCR Primer Design plugin in TBtools (v2.110) (Table S3). Following validation with Oligo 7.0 software, the primers were synthesized by Hainan Nanshan Biotechnology Co., Ltd. China. The expression pattern analysis of *AcHSP70s* was performed using the ChamQ Universal SYBR qPCR Master Mix (Nanjing Novogene Bioinformatics Technology Co., Ltd. China.), following the manufacturer’s instructions. The QuantStudio 1 Real-Time PCR instrument (Thermo Fisher Scientific, Waltham, MA USA) [63] was also used. The relative expression levels of target *AcHSP70s* were calculated using the 2^−∆∆Ct^ method by normalizing internal reference, as described previously [64]. Statistical significance in gene expression levels was assessed using SPSS 16.0 software (*p* < 0.05), while gene expression graphs were generated using SigmaPlot 14.0 software.

## 5. Conclusions

The outcomes of our current study provide the identification of 21 *AcHSP70* genes in the pineapple genome. Our investigation reveals that the pineapple *AcHSP70* genes are classified into four subfamilies. The *AcHSP70* genes transcriptome analysis and expression pattern suggest their diverse role against abiotic stresses in pineapple. Taken together, these findings potentially assist in facilitating further research regarding the evolutionary history and biological functions of the HSP70 gene family and provide essential clues for future research and in-depth identification of pineapple abiotic stress-resistant breeding candidate genes.

## Data Availability

The data are available on request from the corresponding author.

## Supplementary Materials

The following supporting information can be downloaded at: https://www.mdpi.com/article/10.3390/ijms252413407/s1.
