# Supplementary material for: Identification, Phylogeny, and Expression Profiling of Pineapple Heat Shock Proteins (HSP70) Under Various Abiotic Stresses
Source: Int J Mol Sci. 2024 Dec 14;25(24):13407. doi: 10.3390/ijms252413407 (PMC11678451; doi:10.3390/ijms252413407)
Supplement: Supplementary file 1 [file ijms-25-13407-s001.zip › Supplementary Figure captions.pdf]

Figure S1 Distribution of conserved motifs within AcHSP70 family members.
